# Agonist-mediated assembly of the crustacean methyl farnesoate receptor

**DOI:** 10.1038/srep45071

**Published:** 2017-03-21

**Authors:** Elizabeth K. Medlock Kakaley, Helen Y. Wang, Gerald A. LeBlanc

**Affiliations:** 1 Department of Biological Sciences, North Carolina State University, Raleigh, NC 27695 USA

## Abstract

The methyl farnesoate receptor (MfR) orchestrates aspects of reproduction and development such as male sex determination in branchiopod crustaceans. Phenotypic endpoints regulated by the receptor have been well-documented, but molecular interactions involved in receptor activation remain elusive. We hypothesized that the MfR subunits, methoprene-tolerant transcription factor (Met) and steroid receptor coactivator (SRC), would be expressed coincident with the timing of sex programming of developing oocytes by methyl farnesoate in daphnids. We also hypothesized that methyl farnesoate activates MfR assembly. *Met* mRNA was expressed rhythmically during the reproductive cycle, with peak mRNA accumulation just prior period of oocytes programming of sex. Further, we revealed evidence that Met proteins self-associate in the absence of methyl farnesoate, and that the presence of methyl farnesoate stimulates dissociation of Met multimers with subsequent association with SRC. Results demonstrated that the *Met* subunit is highly dynamic in controlling the action of methyl farnesoate through temporal variation in its expression and availability for receptor assembly.

Methyl farnesoate is an acyclic sesquiterpenoid hormone and the unepoxidated form of juvenile hormone III found in insects. Methyl farnesoate is synthesized by juvenile hormone acid o-methyltransferase^1^ and is involved in various aspects of reproduction and development in crustaceans^2^,^3^,^4^. For example, some branchiopod crustaceans exclusively produce female offspring via parthenogenesis under certain environmental conditions. However, in response to environmental cues, that denote pending environmental adversity, maternal organisms produce methyl farnesoate which programs oocytes to develop into males^5^. Further, some environmental chemicals, known as insect growth regulating insecticides (IGRs), can stimulate male sex determination in some crustacean species^6^,^7^.

While environmental cues, such as changes in photoperiod^8^, temperature^9^, or the simultaneous reduction in food availability and high population density^10^,^11^ stimulate male sex determination, exogenous exposure to methyl farnesoate also has been shown to stimulate male sex determination^2^,^6^,^12^,^13^. The highest susceptibility to exogenous methyl farnesoate^2^ and methyl farnesoate agonist^14^ exposure is during oocyte maturation. Exposure during a 12-hour developmental window to concentrations of methyl farnesoate, similar to those measured in hemolymph of crustaceans^3^, resulted in a concentration-dependent increase in male sex determination^2^.

Previously, we demonstrated that methyl farnesoate activated the daphnid methoprene-tolerant transcription factor (Met) in the presence of the steroid receptor coactivator (SRC) ortholog derived from *Aedes aegypti*^15^. Subsequent experiments performed with Met and SRC cloned from *Daphnia magna* and *Daphina pulex* corroborated our earlier results and confirmed the protein complex as a methyl farnesoate responsive receptor (MfR)^16^. Further, several IGRs activated the daphnid MfR in gene transcription reporter assays^15^,^16^, with similar relative potency as observed with the stimulation of male sex determination *in vivo*. These experiments supported Met and SRC as being the receptor protein complex mediating male sex determination.

Although the environmental signals that stimulate male sex determination have been elucidated and the MfR receptor subunits identified, the intra-molecular interactions of daphnid MfR activation remain elusive. We hypothesized that daphnid Met actively contributes to the assembly of the MfR. Evidence for this dynamic role of Met was sought by evaluating the temporal expression of the MfR subunit mRNAs along with subunit protein-protein interactions within the cell in response to methyl farnesoate.

## Results

### SRC cloning

We initially demonstrated that daphnid Met associated with SRC to form the functional MfR using SRC derived from *A. aegypti*. Here, we cloned *SRC* from *D. pulex* for use in our assessment. The derived daphnid *SRC* nucleotide sequence (Fig. S1) was 97.8% similar to the *D. pulex* SRC sequence reported by Miyakawa *et al*.^16^. At 2,357 amino acids the *D. pulex* SRC is much longer than *A. aegypti* SRC and contains an extended C-terminal end. Not surprisingly, the gene is more comparable in length and sequence similarity, 79.7%, to *D. magna* SRC. The deduced amino acid sequence of the *D. pulex* SRC had 29.0% sequence similarity to the *A. aegypti* homolog used to locate SRC in the daphnid genome (Fig. S2). The daphnid SRC contains ten “LXXLL” transcription factor binding domains in the C-terminal. The basic helix-loop-helix (bHLH) domain, has 100% sequence similarity to the sequence deduced in *D. pulex* and in *D. magna* and 52% to *A. aegypti*. The Per-Arnt-Sim (PAS) domain, has 100% sequence similarity to that previously deduced in *D. pulex* and in *D. magna* and 37.5% to *A. aegypti*.

### MfR subunit expression

**Figure 1:**
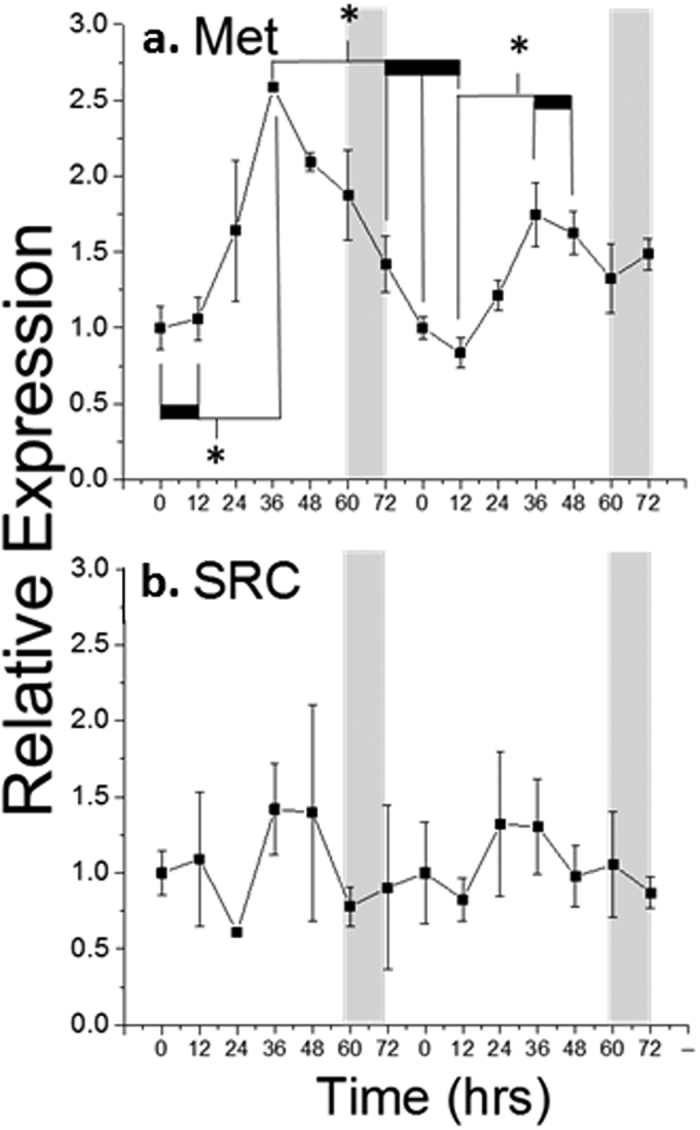
*Met* and *SRC* mRNA levels in *D. magna* over two consecutive 72-hr molt cycles. Each time point was normalized to the initial 0-hour time point. Data are presented as mean and standard deviation (n = 3). Data points connected by a thick horizontal bar are not significantly different but are significantly different from the adjoining data point (P < 0.05, ANOVA). Shaded regions denote the periods of embryo susceptibility to male sex determination by methyl farnesoate.

We hypothesized that daphnid MfR subunits (Met and/or SRC) would be expressed in a temporal fashion such that mRNAs would be present and available for protein production just prior to or during the period of susceptibility to male sex determination. Analysis of *Met* mRNA levels revealed that levels of this MfR subunit transcript oscillates over the course of each molt/reproductive cycle, with base level expression at the beginning and end of each cycle, and peak expression at 36 hours post molt (Fig. 1a). *SRC* mRNA levels did not significantly vary over the course of two reproductive cycles, though expression levels were highest at 36 hours post molt (Fig. 1b). This mRNA accumulation apex occurs approximately 24 hours before the onset of susceptibility to methyl farnesoate.

### Met self-association and dissociation

**Figure 2:**
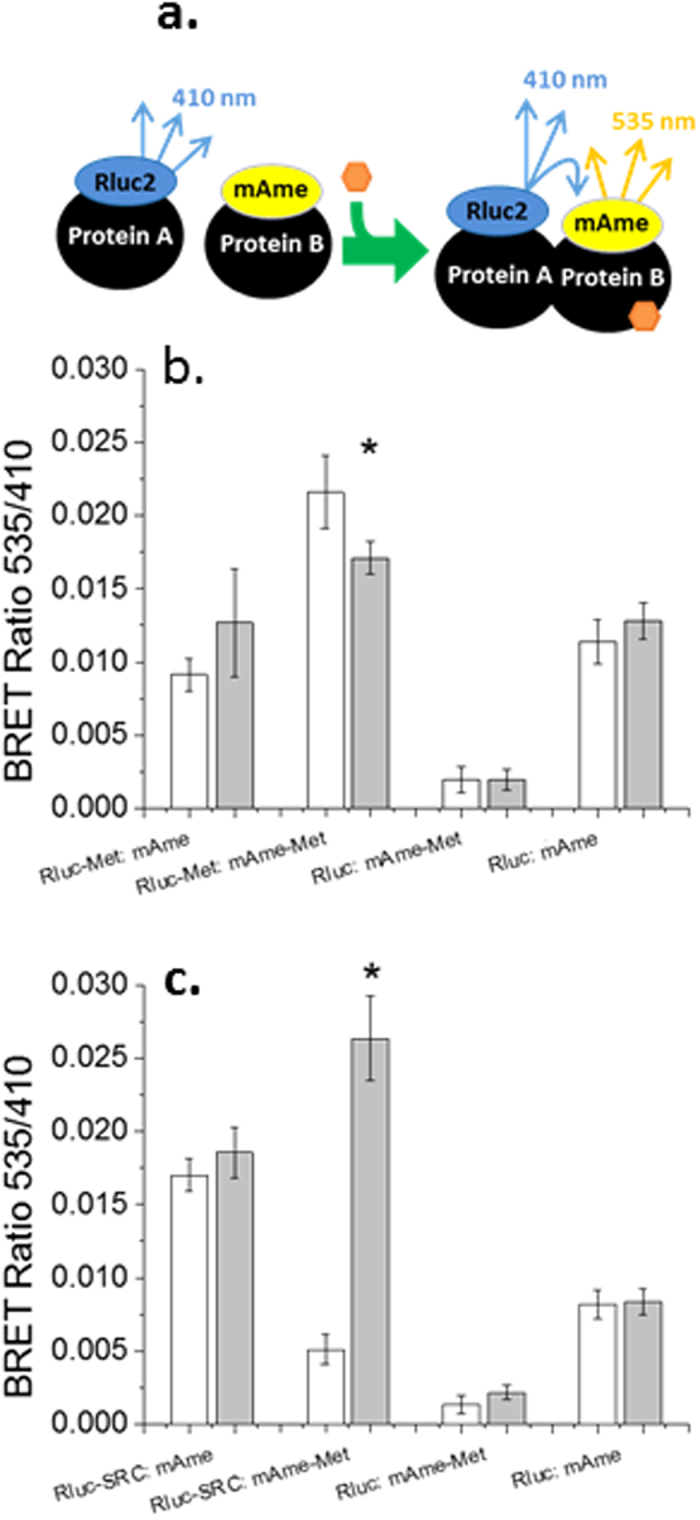
Multimerization of Met and its association with SRC. (**a**) Diagramatic representation of BRET assays used in these experiments. Protein A fused to the photon donor Rluc2 and protein B fused to the fluorophore m-Ametrine are expressed in cells. Photons (410 nm) emitted by the Rluc2 substrate are emitted. Upon addition of agonist (orange hexagon) the proteins dimerize. Now, photons emitted by the Rluc2 substrate excite the closely associated and fixed fluorophore and photons are emitted at 535 nm providing an elevated BRET ratio (535 nm emission/410 nm emission). When assessing ligand-mediated dissociation, an elevated BRET ratio exists in the absence of agonist and decreases with agonist addition. (**b**) Spontaneous multimerization of Met with ligand-dependent dissociation. White bars denote BRET ratios in the absence of methyl farnesoate. Gray bars represent BRET ratios from cells treated with 30 μM methyl farnesoate. (**c**) Methyl farnesoate-dependent dimerization of the MfR subunits. White bars denote BRET ratios in the absence of methyl farnesoate. Gray bars represent BRET ratios from cells treated with 100 μM methyl farnesoate. All data are presented as the mean and standard deviation (n = 3). An asterisk denotes a significant difference between the control and methyl farnesoate treatment (p < 0.05, Student’s t test).

Some bHLH-Pas proteins have been shown to exist in cells as homo-multimers^17^. BRET experiments were performed to evaluate whether Met self-associated in the absence or presence of its ligand, methyl farnesoate (Fig. 2a). Co-expression of Rluc2-Met along with mAmetrine-Met (mAme-Met) generated a BRET ratio that was significantly elevated as compared to assays performed with various combinations of the Met fusion proteins along with free Rluc2 or mAmetrine (Fig. 2b). Inclusion of methyl farnesoate in the assay significantly reduced the BRET ratio in assays containing both Met fusion constructs, Rluc2-Met (photon donor) and mAme-Met (fluorophore) (Fig. 2b), but had no significant effect on the BRET ratio in assays containing free photon donor or fluorophore.

**Figure 3:**
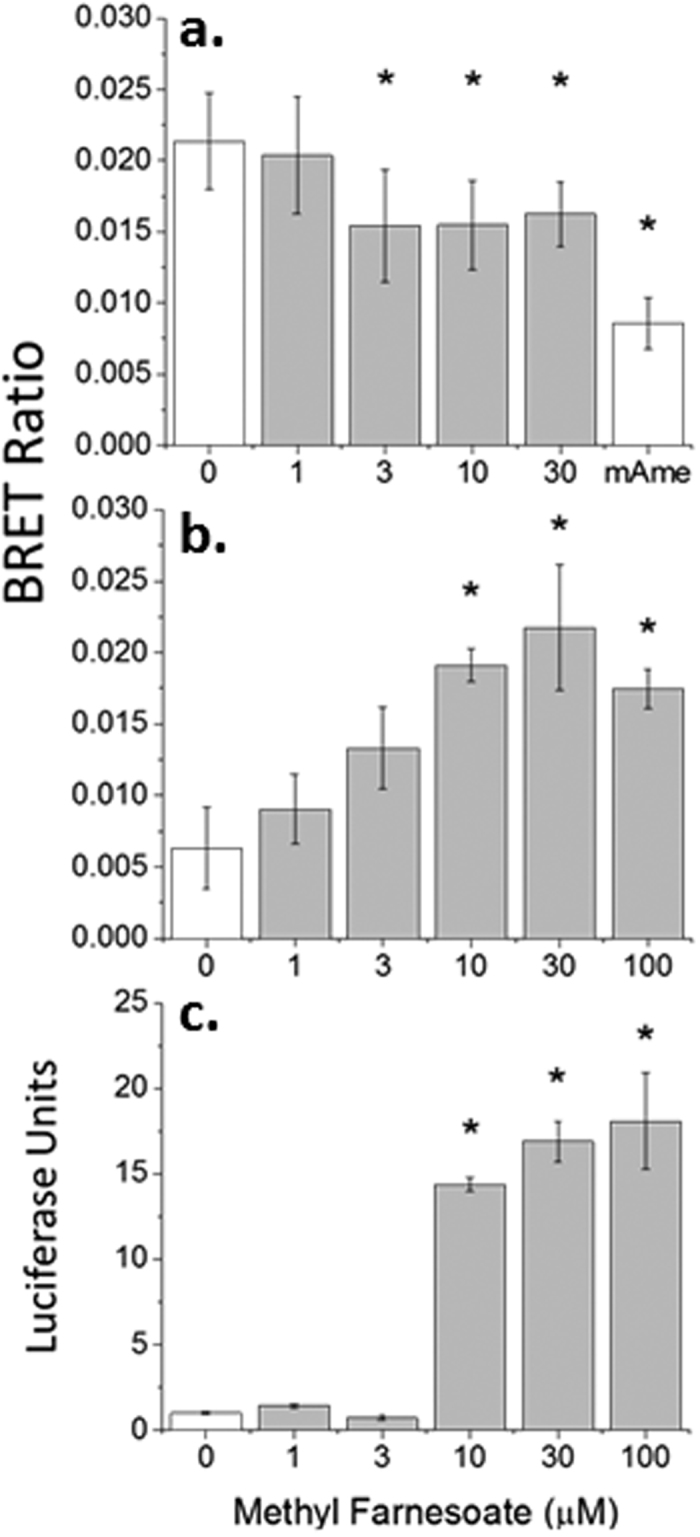
Concentration-responses for methyl farnesoate-dependent MfR subunit interactions. (**a**) Rluc2-Met and mAmetrine-Met spontaneous multimerization and dissociation with methyl farnesoate addition. The treatment denoted as “mAme” consisted of cells expressing Rluc2-Met and free mAmetrine. This treatment provides the BRET ratio that is Met multimerization independent (e.g., baseline BRET ratio). (**b**) Methyl farnesoate-stimulated dimerization of Rluc2-SRC and mAmetrine-Met. (**c**) Luciferase reporter gene activation in cells expressing Met and SRC-Gal4 with increasing concentrations of methyl farnesoate. All data are presented as mean and standard deviation (n = 3). An asterisk denotes a significant difference from the 0 μM methyl farnesoate treatment (ANOVA).

Concentration-response analysis revealed that the BRET ratio generated from cells with both Met fusion constructs (photon donor and fluorophore) decreased in response to increasing concentrations of methyl farnesoate, with a maximum decrease at 3 μM methyl farnesoate (Fig. 3a). The decrease in BRET ratio, in response to methyl farnesoate, represented an approximately 50% dissociation of the Met proteins. Thus, Met proteins exist in cells as homo-multimeric complexes and the Met agonist methyl farnesoate stimulates the partial dissociation of these complexes.

### Met and SRC association

BRET assays performed with cells co-expressing various combinations of Rluc2-SRC, mAmetrine-Met, and free Rluc2 or mAmetrine all produced measurable BRET ratios. However, methyl farnesoate significantly increased the BRET ratio only in assays containing Rluc2-SRC along with mAme-Met (Fig. 2c). These results supported the hypothesis that methyl farnesoate stimulated the association of Met and SRC to form an active transcription factor.

Concentration-response analysis with the mAme-Met and Rluc2-SRC constructs confirmed that methyl farnesoate stimulates Met and SRC association, with significant association beginning at 10 μM (Fig. 3b). Therefore, methyl farnesoate stimulates both the dissociation of Met homo-multimers and the formation of Met:SRC dimers.

### Met:SRC ligand-mediated transcriptional activation

Lastly, we evaluated the ability of the cloned Met and SRC proteins to initiate gene transcription in response to methyl farnesoate. These experiments were performed as we previously described^15^ but with the daphnid SRC in place of the *Aedes* SRC. The reporter gene activation increased with increasing concentrations of methyl farnesoate, with significant transcriptional activation at concentrations ≥10 μM (Fig. 3c).

## Discussion

Environmental cues, such as photoperiod, temperature, population density and food availability alter reproductive patterns in some crustacean species^5^,^8^,^9^,^11^. The hormone, methyl farnesoate, is recognized as mediating many of these actions^18^. The recent identification of the MfR^15^,^16^ enabled further elucidation of how methyl farnesoate controls reproduction and development.

We previously identified the MfR using the SRC ortholog derived from *A. aegypti*, and determined that methyl farnesoate activated Met-mediated gene transcription in the presence of the insect SRC^17^. However, cloning *SRC* from *Daphnia sp* was essential to more fully understand the interaction between methyl farnesoate and the receptor complex. For example, with only 37.5% sequence similarity in their PAS domains, the daphnid and mosquito SRC may have interacted differently with the daphnid Met subunit. PAS domains play an integral role in heterodimerization of PAS proteins^19^, and upon mutation of these domains those protein-protein interactions are diminished^20^.

SRC family members can function as DNA-binding receptor partners or receptor co-activators. The SRC ortholog in *A. aegypti* was shown to function as a DNA-binding partner of *A. aegypti* Met to produce the juvenile hormone-responsive transcription factor^21^. As the *A. aegypti* SRC ortholog activated reporter gene transcription in combination with the daphnid Met^15^ and this activity mimicked the activity of daphnid SRC with daphnid Met (this study), we surmise that SRC also functions as a DNA-binding partner to Met in daphnids and likely other crustaceans.

Members of the SRC family typically house 4–6 “LxxLL” binding motifs (where “L” is leucine and x represents any amino acid), that are responsible for binding of the SRC to ligand-bound partner receptors^22^ and transcription coactivator recruitment^23^. Seven LxxLL motifs exist in the daphnid SRC, while the *A. aegypti* FISC contains only one. The presence of these binding domains may be responsible for the dramatic increase in methyl farnesoate-mediated transcriptional activation (18-fold), compared to that measured in previous assays using the shorter *A. aegypti* FISC (9-fold)^15^.

The relative accumulation of *SRC* mRNA did not significantly change over the course of the daphnid molt cycle, conceivably due to its probable involvement in other reproductive^24^ and metabolic^25^ functions. However, daphnid *Met* mRNA oscillated over the course of each molt cycle, peaking in expression at 36 hours post molt, just prior to the window of oocyte susceptibility to methyl farnesoate^2^. We postulate, that the increased level of *Met* mRNA results in the accumulation of Met protein during the developmental window of susceptibility to methyl farnesaote resulting in the programming of oocytes to develop as males^2^.

Some of the protein-protein interactions between subunits of the orthologous juvenile hormone receptor, in insects, have been characterized. For example, *Drosophila melanogaster* Met proteins form spontaneous homomultimers^26^,^27^ that dissociate in the presence of juvenile hormone^27^ and juvenile hormone analogs^26^. Juvenile hormone binds with high affinity to the PAS ligand-binding domain of Met in some species^26^, and activates the functional juvenile hormone receptor to initiate gene transcription of some developmental genes^28^,^29^. Results from the present study suggest that daphnid Met also accumulates in cells as homo-multimers, although we cannot exclude the possibility that the observed Met complexes were a consequence of overexpression of the protein in our experimental system.

BRET analyses revealed that daphnid Met forms homo-multimers that partially dissociate in the presence of methyl farnesaote. Although the level of dissociation never exceeded 50% even with the addition of increasing concentration of the hormone. This partial dissociation of Met multimers may reflect the dissolution of inactive Met complexes (e.g., quadrimers) to hormone-activated complexes (e.g., dimers). Many bHLH-PAS proteins operate as heterodimeric protein complexes^30^, although transcriptionally active homodimeric bHLH-PAS protein complexes have also been reported^31^.

BRET assays also revealed that methyl farnesoate stimulated the association of Met with SRC. The ligand-stimulated dimerization of daphnid Met and SRC is consistent with the reported dimerization of mosquito Met and FISC (SRC ortholog) in the presence of juvenile hormone^28^. We and others have shown that SRC is necessary for the activation of some receptor proteins^16^,^15^,^22^,^32^.

**Figure 4:**
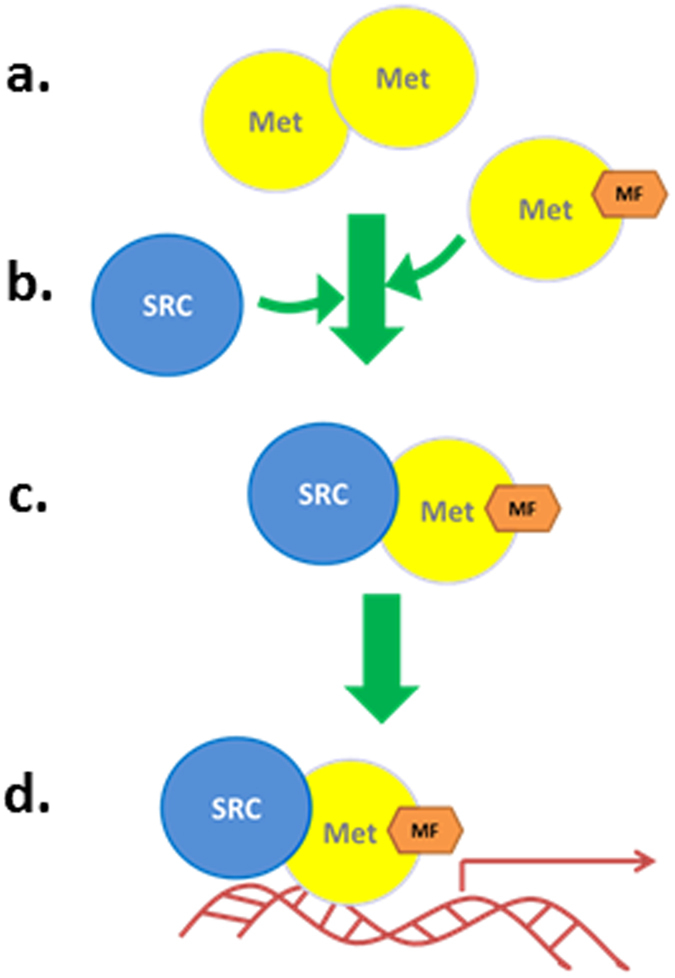
Proposed model for the activation of the MfR by methyl farnesoate. (**a**) Met is rhythmically produced and accumulates as multimers which (**b**) dissociate upon binding methyl farnesoate. Methyl farnesoate-bound Met then dimerizes with SRC (**c**). This activated complex serves as a transcription factor to regulate responsive genes (**d**).

We hypothesized that daphnid Met actively contributes to the assembly of the MfR. Results support this hypothesis. Firstly, Met mRNA accumulates in cells, presumably to provide ample protein, just prior to the period of sensitivity to the Met ligand, methyl farnesoate. The resulting Met protein accumulates in cells as multimers (Fig. 4a), that dissociate in response to methyl farnesoate (Fig. 4b). Upon dissociation, hormone-bound Met binds with SRC (Fig. 4c), and this complex functions as the active MfR transcription factor (Fig. 4d). All measured Met responses to methyl farnesoate (Met dissociation, association with SRC, reporter gene activation) occurred in the range of 3 to 10 μM methyl farnesoate. Methyl farnesoate levels in various crustacean species have been measured to range from 4 nM to 4.0 μM^33^,^34^,^35^,^36^ with the range likely reflecting the nadir and apex of methyl farnesoate production. Thus, the concentration of methyl farnesoate required to activate this signaling event in our experimental system seems biologically relevant.

The identification of the early responses of Met to methyl farnesoate enhances our basic understanding of hormone-receptor interactions in crustaceans, an economically and ecologically important genera. As methyl farnesoate is a critical regulator of crustacean reproduction and development, an understanding of the molecular actions of the hormone may lead to strategies for the enhancement or sustainable maintenance of crustacean populations.

## Materials and Methods

Methyl farnesoate (Echelon Biosciences Inc., Salt Lake City, Utah), was dissolved in DMSO for delivery to the assay solutions. Final DMSO concentration in all assay solutions including controls was 0.001% v/v in BRET assays and 0.0005% v/v in luciferase reporter gene assays.

### Cloning of SRC

*SRC* was cloned from tissues of *Daphina pulex* (clone Busey16, provided by Dr. Jeffery Dudycha, University of South Carolina, USA). Daphnids were cultured in incubators set at 20 °C and a 16:8 hour light/dark cycle. The daphnids were maintained at a density of 20 daphnids in 40 ml of media and were fed once daily with 1.4 × 10^8^ cells of algae (*Pseudokirchneriella subcapitata*) and 0.4 mg (dry weight) Tetrafin^TM^ fish food suspension prepared as described previously^37^. Under these conditions, cultured organisms were exclusively female and reproduced parthenogenetically.

The *A. aegypti FISC* nucleotide sequence (ABE99837) was used to search for the daphnid SRC in the wFleaBase: the Daphnia Genome Database (http://wfleabase.org). Total RNA from adult female *D. pulex* was isolated using the SV Total RNA Isolation System (Promega). RNA integrity was verified by agarose gel electrophoresis (2.0%), and purity by the 260/280 nm ratio. Forward (5′-GGGATTCTAAAACAAAATTGGTACC-3′) and reverse (5′-GAGTCAAGGTCTTGGTTGGATTC-3′) oligonucleotide primers were designed to amplify the entire daphnid *SRC* open reading frame. Amplification of the daphnid *SRC* was performed with an iCycler Thermal Cycler (Bio-Rad, Hercules, CA) using 0.25 U Phusion Hot Start DNA Polymerase (New England Biolabs, Ipswich, MA), 5 μl of 5x Phusion GC Buffer, 0.75 μl DMSO, 200 μM dNTP, 0.4 μM primers, 50 ng template cDNA in 25 μl total. PCR conditions consisted of an initial denaturation at 98 °C for 30 sec, followed by 40 cycles of 10 sec at 98 °C, 30 sec at 58 °C, and 4 min at 72 °C, followed 5 min at 72 °C for final extension. Amplified DNA fragments were cloned into pCR-XL-TOPO vector (Invitrogen, Carlsbad, CA) following the manufacturer’s protocol. Plasmid DNA was sequenced by Life Technologies-ThermoFisher Scientific. The deduced *D. pulex* SRC amino acid sequence was aligned to *A. aegypti* FISC (UniProtKB: Q1KML9), *D. magna* SRC (UniProtKB: M1UYR5), and the recently cloned *D. pulex* (UnipProtKB: M1VDR2)^16^ using Clustal Omega (http://www.ebi.ac.uk/Tools/msa/clustalo/). Putative bHLH and PAS domains were determined by their 100% sequence similarity to corresponding domains in both *D. magna* and the previously cloned *D. pulex*.

### MfR subunit expression

Three hundred adult female *D. magna* were reared as described by Hannas *et al*.^37^ for use in MfR subunit gene expression analysis. Daphnids were kept individually in 40 mL daphnid media and sampled in triplicate where each replicate contained 5–10 daphnids. Beginning at 0 hrs post-molt, daphnids were sampled at defined times over two molt cycles. Replicates were kept in RNAlater^®^ at 4 °C for 24 hrs, then stored at −80 °C. Whole animal tissue was homogenized using a Next Advance Bullet Blender^®^, and RNA isolation and reverse transcription was completed as previously described^37^.

Oligonucleotide primers were designed to measure relative amounts of each MfR subunit (*Met* and *SRC*) mRNA over the course of the daphnid molt cycle. Using forward (5′-CGTGACAAGCTCAATGCCTA-3′) and reverse (5′-GGCTTCATTCGAAGATCCAC-3′) primers, a 149 base pair sequence was amplified from the daphnid *Met* bHLH DNA-binding domain^16^. Another forward (5′-TGTCGCAGATCAACAAGTGTC-3′) and reverse (5′-CGCCAGCTCTTCAATGTAAAC-3′) primer set amplified a 74 base pair sequence derived from the conserved daphnid *SRC* bHLH DNA-binding domain^16^. Amplicon identity was confirmed by cloning and sequencing (Eton Bioscience, Inc.).

*Met* and *SRC* mRNA levels were quantified using 7300 Real Time PCR System (Applied Biosystems, Foster City, CA) and amplification mixtures consisting of 12.5 µL 2x SYBER green (ThermoFischer Scientific), 300 nM primers, 500 ng DNA in a total volume of 25 µL. Reaction mixtures were heated to 95 °C for 5 min, followed by 40 cycles of 95 °C for 5 sec then 60 °C for 1 min. Mixtures were then heated to 90 °C for 15 sec, cooled to 60 °C for 1 min, reheated to 90 °C for 15 sec and re-cooled to 60 °C for 15 sec. A single melting peak was detected for each sample, indicating amplification occurred only for the target DNA sequence. The comparative C_T_ method (2^−ΔΔCT^) was used to assess relative levels of *Met* and *SRC* mRNA (normalized to levels of actin and gapdh within the same cDNA sample). *Met* and *SRC* mRNA levels were normalized to the mRNA levels measured in organisms at 0 hr.

### Fusion protein construction

The association of Met and SRC was assessed using bioluminescence resonance energy transfer (BRET) methodology. Daphnid SRC was fused to the *Renilla* luciferase 2 protein (Rluc2), which served as the photon donor during BRET (substrate: coelenterazine 400 A, emission: 410 nm). The daphnid *SRC* gene was amplified (with a stop codon) from the TOPO cloning vector using primers harboring AgeI (forward) and BstBI (reverse) restriction enzyme sites, and sub-cloned into the pMT-B vector (ThermoFischer Scientific). *Rluc2* (a gift from Dr. Sanjiv Gambhir, Stanford University, School of Medicine, Stanford, CA) was amplified from its original storage plasmid (pcDNA) using primers harboring XhoI (forward) and BstBI (reverse). The reverse BstBI primer also contained a short 24 nucleotide “linker” sequence (AGCGGAAGTGGTAGCGGAAGTGGC) to lengthen the distance between the two proteins and decrease probability of incorrect folding. The *Rluc2*-linker sequence was sub-cloned at the 5′-terminus of the pMT-*SRC* plasmid, to create pMT-*Rluc2*-linker-*SRC* (referred to as Rluc2-SRC).

The previously cloned *Met*^15^ was fused to yellow fluorescent protein *mAmetrine (mAme*) to serve as the fluorophore during BRET (excitation: 410 nm, emission: 535 nm). The *Met* gene was amplified (with a stop codon) using primers harboring NotHFI (forward containing linker sequence, (ATAGCGGAAGTGGTAGCGGAAGTGGT) and BStBI (reverse) restriction enzyme sites, and sub-cloned into the pMT-B vector. *mAme* was amplified from pBad cloning vector using primers harboring KpnI (forward) and ApaI (reverse) restriction enzyme sites, and sub-cloned at the 5′-terminus of the pMT-*Met*, to create pMT-*mAme*-linker-*Met* (referred to as mAme-Met).

Met was also fused to Rluc2, for use with mAme-Met, to assess spontaneous association and dissociation of Met multimers. The *Met* gene was amplified (with stop codon) using primers harboring NotHFI (forward containing linker sequence, AGCGGAAGTGGTACCGGAAGTGG) and BstBI (reverse) restriction enzyme sites, and sub-cloned into the pMT-B vector. *Rluc2* was amplified from pcDNA storage vector with primers harboring KpnI (forward) and EcoRI (reverse) restriction enzyme sites and sub-cloned at the 5′-terminus of the pMT-*Met*, to create pMT-*Rluc2*-linker-*Met* (referred to as Rluc2-Met).

### BRET

BRET assays were performed in *Drosophila* Schneider (S2) cells (Invitrogen). Cells were grown in Schneider’s medium (Gibco, Carlsbad, CA, USA), containing 10% heat inactivated fetal bovine serum (Gibco), 50 mg/ml penicillin G (Fisher Scientific, Pittsburgh, PA), 50 mg/ml streptomycin sulfate (Fisher Scientific) and incubated at 23 °C under ambient air atmosphere. Cells were seeded at a density of 3 × 10^6^ in 35 mm × 6 well plates and transfected 24 hours after plating.

Transfections were performed by calcium phosphate DNA precipitation with the relevant plasmids. Total DNA transfected was constant across treatments, while the donor: acceptor ratio was held at an optimized ratio (producing highest energy transfer), 1: 6 (*Rluc2*-*SRC/mAme*-*Met*). Transcription of the transfected genes was induced with CuSO_4_ (500 μM). Twenty-four hours later, cells were treated with methyl farnesoate for 1 hour in phosphate-buffered saline. Coelenterazine 400 A (5 μM) was then added and light emission was measured immediately at 410 ± 40 nm and 535 ± 15 nm using a FluoroStar fluorimeter (BMG Labtech). The ratio of light emitted at 535 nm/410 nm (corrected for basal level donor emission of Rluc2^38^,^39^) was termed the BRET ratio. The BRET ratio was indicative of the level of dimerization between photon emitter-fusion protein and the fluorophore-fusion protein.

### Luciferase reporter gene assays

Luciferase-based reporter gene transcription assays were conducted to assess the ability of the activated Met:SRC to initiate gene transcription. S2 cells were transfected with plasmids containing Met fused to the Gal4 DNA binding domain^15^, *SRC, Renilla luciferase* (pRL-*CMV*, internal transfection control, Promega) and the reporter gene vector (pGL5-Luc, Promega). Following transfection, transcription was induced with CuSO_4_ (500 μM for 24 hours). Cells then were treated with methyl farnesoate in Ex-cellTM 420 insect serum-free medium with L-glutamine (SAFC Biosciences, Sigma, St. Louis, MO). Cells were harvested after 24 hours of incubation with methyl farnesoate. Firefly and *Renilla* luciferase activity were assessed using the Dual-Glo^®^ luciferase system (Promega) and manufacturer’s protocol. Firefly luciferase activity was normalized to *Renilla* luciferase activity, and each methyl farnesoate treatment group was normalized to DMSO control treated cells.

### Statistical analysis

Significant differences between data points were evaluated using Student’s t test (p < 0.05) or a one-way analysis of variance (ANOVA) followed by a Tukey’s multiple comparison procedure (p ≤ 0.05), as indicated. Statistical analyses were performed using Origin software (OriginLab Corp., Northhampton, MA).

## Additional Information

**How to cite this article:** Kakaley, E. K. M. *et al*. Agonist-mediated assembly of the crustacean methyl farnesoate receptor. *Sci. Rep.* **7**, 45071; doi: 10.1038/srep45071 (2017).

**Publisher's note:** Springer Nature remains neutral with regard to jurisdictional claims in published maps and institutional affiliations.

## Supplementary Material

Supplementary Information
