# Supplementary material for: Agonist-mediated assembly of the crustacean methyl farnesoate receptor
Source: Sci Rep. 2017 Mar 21;7:45071. doi: 10.1038/srep45071 (PMC5359607; doi:10.1038/srep45071)
Supplement: Supplementary Information [file srep45071-s1.pdf]

Supplementary files for:

Agonist-mediated assembly of the crustacean methyl farnesoate receptor

Elizabeth K. Medlock Kakaley, Helen Y. Wang, and Gerald A. LeBlanc*

Department of Biological Sciences

North Carolina State University

Raleigh, NC 27695

Figure S1. ***D. pulex SRC*** **open reading frame nucleotide sequence.**

>Dpulex-SRC (Open Reading Frame)
ATGCTGACGGATACCGCATTCCTGGATGATGCACAGAGTTTGGGTGCAATTCCAT
GCGAGTCCCTATCGTCCGAGCCGTGCTGGGCCAACATGAACACGCTCAGCAGCG
GCGGCGGAGGTGGTGGTGGTGGCAGCAGCAGCAACAGCAACAGCCCCGGTCTC
GGCAACAACAACAATACGGCGTCGTCTTCACCAACGACGACCAACAACAACAAC
AACAGTACTAGCAGTGGTGGTGGTGGTGGTGGCGCTGGCGGCAGCAACGGCCTC
CTCCACGGTCCCAGCGCCTCTGCGGGCGCCATCAAAAAGCGACGGAAATCCGAC
ACGAAGCCGCTGTCGCAGATCAACAAGTGTCTCAACGAAAAAAGACGCCGGGA
GCAGGAAAATGTTTACATTGAAGAGCTGGCGGAATTGATTTCAGTCAGTATAGC
CGACGTCAACTCGCTGTCGGTGAAACCGGACAAGTGCGCCATTTTGCAGGAGAC
GGTCAACCAGATCCGGAAGATCCGCGAGCAGGAAGAAGATGGACGGAGTTCGA
GCAGTAGCAGCAGCAGCAGCAGTAGTAGCAGTTCAAGTTCTAGCAGTTCCGGCA
CCAGTTCTGGGCACAACAACAACAACAACACGACGTCACCCAGCGCTGACGGAG
GCGTTGGTGGCGGTCCGTTGCTCCAGCAAGGCGACGTCTCGTCGTCGAAACCGG
CCCTGCTGGACACGCAACTCTTGGGGACGTTTCTGCTGGAAGCCCTCGACGGCTT
CCTGTTTGTCGTCAACACGGAAGGCAAGACGGAATACGTTTCGGAAAATGTCGC
CCATTTCCTACACTACCAGCCGCAAGATCTCGTGGGCAAATCCATCTACAATTTC
ATCCATCACGGAGATCACGCCCGATTTTCTTCGTCGCTTTTGCCCACGGCCATTG
CTTGGCCGAGTGAAATGGCGCCGGCGTCGCAGAACCGATTGGGCCGCTGTTTCA
ACTGCCGACTGCTGATCCAGCCGCTGGGCGAGCAAGATGAGACGATGGAGGAA
AAGCAGCAGCGAGTCGAGCATTACGAGAACATGCAGATTTCAGCCGTCCTCCAA
CCGTATCCAGGTGATCCAGGCGGTGGCGGCGGCCACCAGCCCAAGAGAAACAGC
GGAGCAGCAGCTGCGGCGGCCGCTGTGGGTTTGGAGGCTTCCGACTTGGAAATG
GCCCTGACCTCTGTGGCTAGCGGCTCCAGCGGAGCTGATCCACAACACTGCCTCG
TCTGTGTCGCCAGGCGGATTCCGTCGACGGAGAAAATGGCGTCGTCGGCCATCG
TCACGACCGGAGGACCCGTCGTCGAGCAGTTCACCACCAAACTGGACTCGACTG
GCAAAATTGTGGCCGTCGACGTCACCGGAGTCTCTCCGCCCTACAGTTCCTACTT
CAGCAAAGAATCCCTCCTCTCGTGTACCATCCAGGAATTGTGCCATCCGGACGAC
TTGTCGATTTTCCAGGCCCATTTCCAGGAAACGATCCAGTCCGGCTGCGGGCTCA
GTTCCAGGTACCGTCTCCGATTGGCCGGCGTCGCAAGTTTCCTGGTCGTCCAGAC
AAAGTCGAAGCGCTTCGTCAACAGCGACACGCACGACACGGATTTTGTCATGGC
CACCCATTCCATCATCGTGGACGACGAGGACGGCCAGGCTGACGGCGGCGGTGG
CCGGTTGATGTTGTCGAAAAGCGACACGCTCAAAGACGTCCATCAGCAGATAAC
GGACGCTGCCAACCAGATGAGCCAACAGCAGCAGCAGACGACTGTCAATCCGGT
GCTGACGAGCGTCGTCCGCCACGATGTGATATCGGCGACCAGTTACGGCAACGG
CCGCAGTGGCGCCACCACCACAACCACTTCCTCCTACTCCAGTACCTTCGCCGGA
TTAAGTTTGGGAACGAGCGGCGATCTTCTCAATGACTTTGTCGTGCCCGACCTCT
TCATGGCTTCGCCTCCGTGGGGTGGCGATTTCAACACCAGCGAGTCCAGCCTGGA
CGGCAACGGACCGCCCGGCGGTGCTGTCGCTTCTGCCGTCGCTTCTGCCGCTGCT
CAAATGAACAACAGTCACCACCACCTGCCGGCCATCGCCACTTGCAATCAGCAA
CAACTCTCGGCTCCATCTCCTCTTTCCCTCGTCCCGTCCGGCGGACCCCTCCTGGC
CGGACACATTCCCCATTCCGTTGGCGGGGGAATGATCAACTGGGGAAGTCCTCC
GCCTTCGGGCGGACAACAACAACAACGGCCCGGCTCCACTTGCTCGGCCCCTCC
CGTCTCGTCGAGGCCCAGCTCGCGTCAGAGCGCTTCATCCACACCGCGACCGCCC

AGCGTTTCGTCAGCCTTCAGTCCGGCTCCCAATTCCGTTTTGGGCGTCGTGCATC
CCTCCCCCGTGCTCAGTCCAGTTGCCACTAGTTTGCATCCGGCCGGAATGATGAG
CTCGGCCACGGCCACCACTTCGTCGTCGTCGTCATCGGCCGGCCAACAGCAACCC
AGTCCGGCCAGCATGACGACGCCCTTTAGCAACAATTTCCCCTTCAGTCCGCTCC
AAGATCCCATTCCTCAGCATCCGGCCACGGCTCCGCTTTCCCTCAACAATCCGGG
CACGCCATTCCCGGATGACCCGGCACGAGATTCCAAGGAAGGAATTCTCTCGTC
CAGCATGGCCACCGCCGCCGCCAATCACGGTCATTATCCGGCCAGCGGTGGAGG
ATCTCACCACCACCATCCCAATCATCACGGGACGCCGGACAAACAACTCAGCGC
TCTCAGCAGTTTGTTGAACAGTAGCGACGCCTCAGCAGCAGCAGCAGCGTCGTC
GTCTGTGACGGAATCGGGGCGGCTGCGCATTTTGCTCATGCAGAGACCGGGCAA
CGGGCCGCCAGCTCCACCGTCGGCTGGCCATCTTTCGACTAGTATCAACGGGAG
CCATCTGTTGAGTAGTTTGGGCAGCGACAGCAGCGACGGCGTCAAACGTGAGAA
GGAGGAGACTAGCAGCAGCAGCGTCGGTGGCGTCGCCTGTGGCTTACAGCTGGC
CGGAAGCAAAGGCAACCACGACAACCGCATTCTCAAAGGCCTGCTGAATCAGGA
CGATGGCGACGAAACCGATCCGGCGGATGAGGGCGGCAGCGGCAGTCATCGCTT
CTTGCTGGCGGGCAGAGGCAACTCGGGCGACAACAAGAGCAGCTCCGTCAACAA
CAATAACAACAACAACAACAACATGCTGCACAAGCTGCTGAACGTGCGGAGTGA
TGACGATGCCGAGCAGCGGATGGGTCTGCGCAAGCCCAACGAATTGCTGAAAAA
ACTCCTCAAGGATTCCGACGAGGATCATCAACAGGCTGGTGGTAGCGGCAGTGC
TTGCGGTGGCGCCGGAAGCAGCAACAACACGGCCAGCCAGCAACAACAACACC
ACCACCACCCCATCCAGCAGCAGCAGCAACAACAACATCCCGATCAGGTGTCGT
TCCAGGAAGAGCAATTACTCAAGTCGTTGGGCTTCCCATCGCCGACAACGTCATC
AGGTTCGTCTACGACTCCAACGGTAACAACGGGATCGTTGGCCAACATGTTGAG
TACCAGTCAGACCACTCACCTCAAGTCTCCACCGCCAGGCATGGCCAGCCATTGC
GATGGATTGCTGGAATTTGGCGGAGTCGTTTGCAGCAGCACGACCAGCAGTGGG
CTGCTCTTTGGCGGCGGGCCGGTGGGTGGTGGTGGCATGCGGGGCACCAAGAGA
CACAGCGAAGAAGCCCGCGACGAGGTGAAGGCCAGCAAGGAGCCCATGCTCAA
CGCCGACCACCTGATGATGTCCGCAGGCGGAGGTGGGGTCTTGCATCAACTGCT
GGGTCCTCCAACGTCATCGTCGTCATCCTCTTCCTCATCTTCGACATCAACGAAT
CCGCACAGCAGCACAGTGGCAGCTCCTCTGTCCGTCGGCTCATTGCAACATCAAC
CGCTTTCATCGCCAGCAGCTGCCGTTGCGGCCGCCACCTCCGTCGTGGCTGCGGC
GGCCGCCACAGCCAACATTGCTGCCGCTTCGGCCATCCAGTCAGCCGCTGCTGCT
GCCGCTAATCTCCCGGCCAGCAGTAAATTGTGCGAGAAGAATAAGATGTTGGCT
TCGTTGCTGGCCAAGACGCCCGTCATCCCGTCACACTCGCTGTCGACGAGTATCG
CCTCGCCCAAACCGTCAGCGTTGCCTCAGGAGAAATTGCCCAAGGACTTGAAGG
AGAAAATCCTTCAGACGCCGCCAGTGAGTGGAACGTCGGCTCCGGGCGCCCATT
GGGCCGGAGGCTCGGTGCAAACCCAGCCCTCGACGACTATGCAACTGCCCCCGC
ATCCGCCAATGCAACAACCCCAGCAACAGCAGCAGCAACAACAACAACACCTTT
TGAACCAACAGCAACAACAACGACATCCGGCCATGCAACAGTCACAGCAGCATC
AGTTACACCCCCGATCTCAGCACCCACAACAGCAGCAGCAGCAACAACAACAAC
AACAACATTTGAACCTCAGCCAGAAACAGCAACAACAACCAGCTCCTCAGACTG
GAGGCTTTTTGAATTCTCTTTTGAATCGGCCCATTGATTCGGCTTCCGGTCCCAAT
CTGGGCGAATCGCAACAGCAACAACAGTCTAATCAACAACAGCAACTATATTTA
GCAGCCCAACAACCCCCGCCCCAACTCCAACAACAACACCGACCCAATCAGAAC
CCCGTTCAAAAGCTGTCTCTCAACAATCAGCAACAACTTTCGGCCGCCATGAACT
CCTCGGGTGGTTTCAACATTTCCCTTCATCCGCAACAGCACCAACCTCACCACCA

GCAACAACAACCCCAACAACAACATCAACTGCAACATGCAATATCTGGCCCCAT
GGGCCCCGATCGAAGCCTGCAGCAAAGCGGAGGAGCAGGTCAGCGATCCGGAA
TGTCCATCCAGCCGCAACAACAACAACAACAACAGGCTTCGCATTCCAATCCCA
TGTTGGCGGCCACCAGTTTGGAGAGCAGCGACGGCAGTTACAGCGTCCACGCCG
ATTTCATGACTCCGCTGGATGTGCAAATGGCGTCGGTGGCCGGATGGGGTGACA
CGCCTTCGATGGACCCCGAACTCTCTGACATCATCGAGCAAGTCATCGACATGG
ACGAGAGATACGAAAGCGATTCCATGATCTTTGGCGAGCTGACGTCCGCCACGC
CACCGCCGGTGACTATCCAGCCGGTCATGTCCATCGTCCAGTCGTCGCAGAGCGT
CACGTCGGTGACTCAGACGACTGCCGTTGTCGGCCCGTCGGGTCCCCTCCTGCAA
ATGGACATGTCGAAAGAGAAACTGGCCATCACAGCCATTCAAAAGTCGCTCATG
TCCTACGAGAAAATCTCGCCAGTCGCTCAAAGTCCGCCGGCCTACAACCTGCCG
GGCTACGCACAACAACAACAACAGGGTCAAGGAGCGCCGATGAGAATGTCCAC
TCCGTCGTACGGGATGAATTCGCCTTCAGCTTCGGCTGGGTCCATCATCATGGCC
AATCAGCAGCAGCAGCAGCAGGCCAGTCTCACCATCCCCGGTGGCAGAAAGCAG
AAATTGCCCGTCCAACAGCGACGACTCTTGAATCGACAACAACAACAGCAGCAG
CAGCAACAACCACAAGATCAGCAGCAACAGCAGCAGCACCCACCTCAACAGCA
GCTCCTGGGCGTCTTGCTGAACGAATCGTCGGTACAACACACCCAAGGTCCCCA
GCTCTCACCTGGAGCCCTGCAAATTATGGATGACCTACTTAACGCCATCCCACCC
AACATGACTATATCCAGGTCGGATTCACAAGTTTCACCCAACTACGGTTCGGTGA
TGTTGAGCAGTCCACTGGGTAGCGGTGGGCCGCTGGCGTCTCAAATCTCTCCCAG
TCAGCGCCCGATGCAACCCCCTTTTTCGCCTCACGGCCCGATGAACACGGCCAAC
GGTGGCAACTACAATCAAAGCCCTCAGGCACAAATTGTCAATAACAACAGCGGC
AACAACTTCGCCCCCGGCGGACCGTCTTCTCGGCTCTCGCCGTTCAATTCGGCTC
AAGTGTCGCCCCGCATAGGGCCACCTCCGCAAAATTTTCAACAGCAACAACAAC
AGCAGCAGCAGCAACAACAACACGTATCCCAGCTTTCTCCGCAGCCAGGAGTGG
GCGCCAGATCCAGTCCTGGAATCAGTTCAATGCAAGGGCAGCCTAGTCCTGGCG
TTGGGGTAGGATCGTGGAACAACAACATGCCGAATCGTCCTAATTTGCAACCGC
AACAACAACGACAGCAGCAGCAGCAACAACCTCAACAACAACCTCAACAGCAG
CATTTGCTCGGTTATGGACAAGGCATTCGCGGTGGCTACGCTCCTCGACCCGCTA
TGAGAGCATTGCCCAATTCCGGTCCGCCCAACTGCGTCGGCGGACCTGCTGGCCC
GGGCAATAGTAGCAACAACAACAACAACATTCGACAAGGCTACGGTAACAACG
GCATGACCGACAATCCAACCGGACCATCTTCCTCCAACGGGATGTTTGCTCGCCA
GCAACAACAACAGCAGCAGCAGCAGCAACAACAACAGCAGCAACAACAGTTGA
GATTACAGCGAACCGTGAGCGCACCTAGCGGGGTTATTCCAGGTTCGGTCGGTG
GTCCAGTTCAGGGTGGCGGAAGAAATGACGGCGGACCGCCTGGGATGGCCTCCG
AGTACGTTCGCAAGGAGCTGAAAGCGGTGGTAGGAGCCCGAACAATCCAAAGA
GGTTTGTCTGGTCCGGTTGTGAATGTGAACCACGCGTCGGGCCAGATGAACCAC
AGCTCACCTCCCATGATGGTACCGGGTGGTAAGCCACTCAATCAAGCCGATCTTG
AAGCCCTCGGTCTCAGCTACGAATTGCCCCAGGGGAATGACGGATTACACGGAA
GAGTTTGGGATAGTCCCAACATGGGCGAGTCGCCGTCGCAGACAATGCCGTCTA
CCAGGAATACGATGGAAGAAGCTCCGCGGCCAGCTGATCCTCAAATGTCTCTTC
TCAAGCAGCTACTCTCGGAGTGA

Figure S2. Alignment of the deduced *D. pluex* SRC, *A. aegypti* FISC and *D. magna* SRC
The shaded sequences indicate the conserved bHLH and PAS domains, as well as the C-terminal LXXLL domains in the *D. pulex* SRC.

```
D. pulex       MLTDTAFLDDAQSLGAIPCESLSSEPCWANMNTLSSGG-----------GGGG-GGSSS
A. aegyp       -----MSIAAAENAGLGPCELPL-PDHWLVHTQLSQSSSQQYSTPSPAIGGPSLGLGGL-
D. magna       MLTDTAFLDDAQSLGAIPCESLSSEPCWANMNTLSSGG-----------GGGGGSGGSS
                      :  *:. *  ***       *   . **...            * .   *.

D. pulex       NSNSPGLGNNNNTASS---SPTTTNNNNNSTSSGGGGGGAGGSNGLLHGPSASAGAIKKR
A. aegyp       ---PPALPQQQS--SSHSTSPLQQTPSA----SQQQQLQPKIMNAVVPAVSVPAANKKIR
D. magna       NSNSPGLGSNNNNHTTSSASPTTTTTTGNSNSSG---GGAGGSNGLTG---TSAAAIKKR
                   *.* .::.  ::   **   . .     *          *.:     . *.  * *

                      bHLH_________________________________________________
D. pulex       RKSDTKPLSQINKCLNEKRRREQENVYIEELAELISVSIADVNSLSVKPDKCAILQETVN
A. aegyp       RKPDTKPQSQINKCNNEKRRRELENEYIEQLGEFLQINKRDM--TACKPDKAAILSEVVK
D. magna       RKSDTKPLSQINKCLNEKRRREQENVYIEELAELISVSIADVNSLSVKPDKCAILQETVN
               ** **** ****** ******* ** ***:*.*::.:.  *:   : ****.***.*.*:

               _____
D. pulex       QIRKIREQEEDGRSSSSSSSSSSSSSSSSSS---SGT-----------------------
A. aegyp       TFRRLLEQGNRDLTGSNRCSKCSPDCSDSC------------------------------
D. magna       QIRKIREQEEDGRSSSSSSSSSSSTSSNGSSTNSSGTSSANSGTGGAVGAVGAGGAGGAV
                :*:: ** :   :.*. .*..*  .*...

D. pulex       -----SSGHNNNNNTTSPSADGGVGGGPLLQQGDVSSSKPALLDTQL------------L
A. aegyp       -------------------------KLHPVQQGEVSSTEPPLPEPSVNGHSPEKSAYFEA
D. magna       GAVGAVGGGGQGNNTTSPGGVTDGGVGPLLQQGDVSSSKPALLDTQL------------L
                                            :***:***::* * : .:

                  PAS______________________________________________________
D. pulex       GTFLLEALDGFLFVVNTEGKTEYVSENVAHFLHYQPQDLVGKSIYNFIHHGDHARFSSSL
A. aegyp       VKYYISNVGWVLLEINSEGVIECATDNVLDVLHYTRTELHGQSIYSYLHTGDHSKLSPIL
D. magna       GTFLLEALDGFLFVVNTEGKTEYVSENVAHFLHYQPQDLVGKSIYNFIHHGDHARFSSSL
                .: :. :  .*: :*:**  * .::** ..***   :* *:***.::* ***:::*  *

D. pulex       LPTA--IAWPSEMAPASQNRLGRCFNCRLLIQPLGEQDETMEEKQQRVEHYENMQISAVL
A. aegyp       NKNSFELNWDQNEFLQPPKRTIR-TKIRWLLKTPENANDTIEQKQQRQEKYKDLLIISAP
D. magna       LPTA--IAWPSEMAPTSQNRIGRCFNCRLLIQPLGEQDETMEEKQQRVEHYENMQISAVL
                 .:  : * .:      :*  *  : * *::   : ::*:*:**** *:*::: * :.

D. pulex       QPYPGDPGGGGGHQPKRNSGA----AAAAAAVGLEASDLEMALTSVASGSSGADPQHCLV
A. aegyp       VK--DDTD--------------------------------------------AESSSVLCLI
D. magna       QPYPADGGQQQQQPTKKISGAAAAAAAAAAAVGLETSDLELALTSVASGSLAGDPQHCLV
                    *                                              ..   **:

D. pulex       CVARRIPSTEKMA--SSAIVTTGGPVVEQFTTKLDSTGKIVAVDVTGVSPPYSSYFSKES
A. aegyp       TL----PEDEHHQGPAIESHTMPQTLDEQLTLKLDMTGAVIDFDAATLRKQFTDYLTKET
D. magna       CVARRIPSTEKMA--AA-NPTVGGPIVEQFTTKLDPSGKIVAVDVTGVSSPYSSYLSKEA
                :    *. *:    :    *    : **:* *** :* :: .*.: :   ::.*::**:

D. pulex       LLSCTIQELCHPDDLSIFQAHFQETIQS-GCGLSSRYRLRLAGV--------------AS
A. aegyp       VR--SIHDLCHFQDRPRLNEHLQNVMNANGAAQVSSYRLRLGGP--------------DV
D. magna       LVSCTIQELCYPDDLPVFQAHLQETLHS-GCGISSRYRLRLTGASTPMGHRGGAGGGSGG
               :   :*::**: :*   :: *:*:.:.: *..  * ***** *

D. pulex       FLVVQTKSKRFVNSDTHDTDFVMATHSIIVDDEDGQADGGGGR----------L--MLSK
A. aegyp       YVHVKAQTRLFRNDKPNEPDFIMAIHTILNDNEVAMVESGLNNPSSSSSSS--LA-IPS-
D. magna       FLVVQTKSKRFIHGDTHETDFIMATHSIIVDDDGEDVDTGNSSSSNTNTNTSRMMLLAAA
               :: *::::: * . . .: **:** *:*: *::   .: *             :  : :

D. pulex       SDTLKDVHQQITDA---------ANQMSQQQQQTTVNPVLTSVVRHDVISATSYGNGRSG
A. aegyp       -----------TSSGSGSSTARQMQQI-TQGVSNMGGPLMSSIL----------------
D. magna       SDTLNDHHRLSESTGSIISQPQQQQQQQQQQQQQSNPVLTSVVRHDVISATSFGNSGCS
                           .:          :*   *  .    *:::*::

D. pulex       A-------TTTTTSSYSSTFAGLSLGTSGDLLNDFVVPDLFMASPPWGGDFNTSESSLDG
A. aegyp       ---------------------------------------------------NGSGN---S
D. magna       GGATTATTSSTSSSSYSSTFAGLSLGTSGDLLNDFVVPDLFMASPPWD--FNNSESSLDD
                                                                  * * .

D. pulex       NGPPGGAVA-----SAVASAAAQMNNSHHHLPAIATCNQQQLSAPSPLSLVPSGGPLLAG
A. aegyp       GGPGGPSGLQGALGSVVSPRSSN---LHSSL--LSQ------QSSDGSSFFPSEFEFEFP
D. magna       GGSGVALAAASAATSAAVAAAAQIT-NSHHLQAVTATSSLQLSVPSPLSLIPSGGPLLAG
                *            *..   :::       *  ::       .  .  *:.**   :

D. pulex       HIPHSVGGGMINWGSPPPSGGQQQ--QRPGS-TC---SAPPVSSRPSSRQSASSTPRPPS
A. aegyp       HSTYDMESVGVNWDSRPDSRTS---------------------------VTPVSTPRPPS
D. magna       HLPHHSVGGMINWGSPPPSAGAGGVGQRAGSTPCGQMATVMGSSRPSSRQSASSTPRPPS
               *  :   .  :** * * *                               :  *******
```

```
D. pulex       VSSAFSPAPNSVLGVVHPSPVLSPVATSLHPAGMMSS--ATATTSSSSSSAGQQQPSPAS
A. aegyp       VT-AYSPAAASL----CPSPLT--------------------YQ--PSSAGGQ--PSPSN
D. magna       VSSAFSPAPNSVLGVVHPSPVLSPVGATSIHSAAMSCTSGLVTGSTSSSSAGQQQPSPAS
               *: *:***  *:     ***:                          **:.**  ***:.

D. pulex       MTTPFSNNFPFSPLQDPIPQHPATAPLSLNNPGTPFPDDPARDSKEGILSSSMATAAANH
A. aegyp       NQQVN-NN----------------------NNNS-----SMTNNNAGLFG----GFQCQ-
D. magna       MTTPFSNNFPFSPLQDPIPQHPATAPQT-HNPATPFLDE-VRDTKDGIISSGPGGS-VSH
                     **                      *  :        : : *::.        .
D. pulex       GHYPASGGGSHHHHPNH---------------HGTPDKQLSALSSLLNSSDASAA-----
A. aegyp       ---FDD-KDEVQEQIQQQQQQLQQQQQQQQHQQQMASHDSERLRNLLTTKRPHSNASSSS
D. magna       GHNYLSGGSHHQQQHHHH---HQQQQQQQQQQQHATDKQMSALSSLLNCNDAATSSSSSS
                    .     :.: .:               :   .:: . * .**. .   :

D. pulex       -----AAASSSVTESGRLRILLMQRPGNGPPAPPSA--GH--------------------
A. aegyp       ----GMDMDHDHRNPNRILKLLNDKSDDD-DADARNRPSELLRQLQ--------------
D. magna       SSSSSSSSSSSLAESGRLRILLMQRPGNAPPPSAHQQPGHSSSGSSSSSSSTSTSTSTST
                       . .  :  *:  ** ::  :          ..

D. pulex       ---------------------------LSTSINGSHLLSSLGSDSSDGVKREKEETSSSS
A. aegyp       -------------------KVKDEPKPHQAPLKDEELLQMLQVQRNDR-KRSSTEP---D
D. magna       STSTNTNTNTNSSNNNNNNNNNNASGNNNSSSVNGSHLLAALGDA--EAVKREKDENSSA-
                                           .: :: ..**  *     :  **.. *

D. pulex       VGGVACGLQLAGSKGNHDNRILKGLLNQDDGDETDPADEGGSGSHRFLLAG-RGNSG-D-
A. aegyp       EGAAAKRSDDKPSKLREKNKMLASLLANPAKAPTQ--MLAGHSSLRIIPDIPTSNISRQM
D. magna      ---PSCG-SLSVTKGSHGNRILKGLLNQDDGDEADQADDT--SNHRFLLTA-RGNLS-GD
                   :   .   :*  . *::* .** :     ::        .. *::     .* .

D. pulex       -------------NKSSSVNNNNNNNNNMLHKLLNVRSDDDAEQRMGLRKPNELLKKLLK
A. aegyp       GSVTSSTAP----NQTLNNNNLTTSNNN-LKHVQHLRMQQQQ----------QMRKSAMP
D. magna       GVKTSSSAGNNNNNTTNTNNNNTNNNNNMLHKLLNVRSDDDAEQRMGLRKPNELLKKLLK
                            * : . ** ...*** *::: .:* :::           :: *. :

D. pulex       DSDEDHQQAGGSG--SACGGAGSSNNTASQQQQHHHHP----------------------
A. aegyp       SPSQQPP-------------------TSSDIYLSHQQQQQALLQQQL-------------
D. magna       DPEEDHQQASGNGSSSACGGTGSGGTANQLQQFHHQRQQQQQQQQQHQQHQQHQQHQQSQ
               . .::                     : .     *::

D. pulex       -IQQQQQQQHPDQVSFQEEQLLKSL---GFPSPTTSSGSSTTPTVTTGSLANMLSTSQTT
A. aegyp       -IAA-QQRQQNYAAAIQDS-GIGSVGSGPFATPSTAT-------------------STTS
D. magna       QQQQQNQHHHSDQVSFQEEQLLKSF---GFPSPTASSGSSTAATVATGSLANMLATSQMT
                    :*:::   .::*:.  : *.    * :*::::                   *  :

D. pulex       HLKSP-PPGMASHCDG--------------LLEFGGVVCSST------------------
A. aegyp       HATPEWDPELNEILNHVIDIAPDVSFADSELTSFLGSMDPSTSTAPSQASQQDIQEKLAI
D. magna       HLRSPQPPGMSSHCDG--------------LLEFGGVVCSST------------------
               *      * : .  :               * .* * :  **

D. pulex       -------------TSSGLLFGGGPVG--GGGMRG---------TKRHSEEARDEVKASKE
A. aegyp       NAIQKSLMREIENVTSPMQYSGSPPAYPMHGMGSVGSPAGQMSQQTPGTPTTPNPNFTPP
D. magna       -------------TASGMVFGGNAAVGGASGMRG---------TKRHSEEARDEVKASKE
                            .:* : :.*        ** .          :  .  :  : : :

D. pulex       PMLNADHLMMSAGGGGVLHQLLGPPTSSSSSSSSSTST-----------------NPHS
A. aegyp       PVYQPRLRMSGPGGPGVGQLGNGAPSPSA-------------------------------
D. magna       PMLSADHLMMSAGGGGVLHQLLGPPPLSTASTSSSSSASSSSACSSSAALSSSLAPSNAH
               *: .    * . ** ** :   * *  *:

D. pulex       STVAAPLSVGSLQHQPLSSPAAAVA--AATSVVAAAAATANI------------------
A. aegyp       ------IAMQKLSHQQQQR---------------------ERMQEQQRQRLLQQQKQQQM
D. magna       SVAATTLAIAALQHQPLSSPSTAAAVAAATSAVAAAAATANAMASQLQA-APSAQSAAAA
                     :::  *.**  .                      :

D. pulex       ---AAASAIQSAAAAAAANLPASSKLCEKNKMLA-------SLLAKTPVIPSHSLSTSIAS
A. aegyp       V-----VPVNATANADLNLGLTPGMQNIESLLNNTVAPNVSLTRANSVVPDSQLSPGF--
D. magna       VAAAAAAAAAAAAAAAAANLPASSKLCEKNKMLA-------SLLAKTPVMPSPS-STNIAS
                         ::* *  **  :  : : :.:*        **   . *:*. . *  :

D. pulex       PKPSALPQEKLPKDLKEKILQTPPVSGTSAPGAHWAGGSVQTQPSTTM------------
A. aegyp       -SPSQLMQQQLSPNQRTQL---------SPQQTGFQGNPFNNNPGHRMSPQQQQQLVAAA
D. magna       PKPSALPQEKLPKDLKEKILQTPPVSGTSAPGAHWAGGSVQTQPPTTM------------
                .** * *::*  : : ::         *   : : *  .:.:*   *

D. pulex       ----------QL-PPHPPMQQPQQQQQQ------------------QQQHLLNQQQQQRH
A. aegyp       GFQQGNPGNQQLSPRQPPFNTSQQLPQANAAALQQ-----QQQQQWQQNARLS--IQQQN
D. magna       ----------QL-PPHPAIQQPQQQQQHVLNQQQQQQQQQQQQQQQQQQQQQQQQQQQRH
                         ** * :* ::  **  *                   **:   .   **:.

D. pulex      PAMQ--QSQQHQLHPRSQHPQQQQQQQQQQQHLNLSQKQQQQPAPQTGGFLNSLL-----
A. aegyp      PMLNAQLSTVPGFNPAAAANRQF-VAPQRQRSLN------SPGTPRQGSFGSTVDGSGFP
D. magna      PAMQ--QSQQHQLHPRSQHLP------QQHQHLNLGQKQQQQPTPQTGGFLNSLL-----
               * ::   *    :.* :          *::: **      .  :*: *.* .::
```

```
D. pulex       ----NRPIDSASGPNLGESQ--------QQQQSNQQQQLYLAAQQPPPQLQQQHRPNQNP
A. aegyp       GPPSPSQPNAGAGPNFGNPVFAN-------------QQMRLQRQGSVPPQATQHL-----
D. magna       ---NRPIDSASGPNIIGESQQQANQQQQQQQQQQQQQLYLAAQQPPP-VQLQHRPNQ-T
                       ::.:***:                    **: *  *   *    **
D. pulex       VQKLSLNNQQQLSAAMNSSGGFNISLHPQQHQPHHQ------------------------
A. aegyp       ------------------------------------------------------------
D. magna       VQKLSLNNQQQLSAAMNSSGGFNISLHPQQHQQQQQQHHHHLQQQQQQQQHQQQQQQQ

D. pulex       ------QQQPQQQHQLQHAISGPMGPDRSLQ-QSGGAGQR---SGMSIQPQQQQQQQASH
A. aegyp       --------------------PGSPRPSY----GGHGPGPDTSGYGMMFNNATMQQQ----
D. magna       QQQQQQQQQQQQHHQLQHQLSGSMLADRGLLQAGGGPGQR---SGMSIQPQQ----QTSH
                                    *    .      . * *      ** ::

D. pulex       SNPMLAATSLESSDGSYSVHADFMTPLDVQMASVAGWGDTPSMDPELSDIIEQVIDMDER
A. aegyp       --------HSAGSPGDYFN-----------------------------------------
D. magna       ANSMMAAASLESSDGSYSVHADFMTPLDVQMASVAGWGDTPSMDPELSDIIEQVIDMDER
                          .* *.*

D. pulex       YESDSMIFGELTSATPPPVTIQPVMSIVQSSQSVTSVTQTTAVVGPSGPLLQMDMSKEKL
A. aegyp       -RSQAEFFGGNTSR----------------------------------------------
D. magna       YESDSMIFGELTSVTPPPVAIQPVLSV-QSSQAIASVAQATAIVGPLGPHLQMDMSKEKL
                .*:: :**  **

D. pulex       AITAIQKSLMSYEKISPVAQSPPAYNLPGYAQQQQQGQGAPMRMSTPSYGMNSPSASAGS
A. aegyp       ------------------------------------------------------------
D. magna       AITAIQKSLMSYEKIPTVAQSPPAYNLPGYAQ---QGQGAPTRMSTPTYGMNSAPASS--

D. pulex       IIMANQQQQQQASLTIPGGRKQKLPVQQRRLLNRQQQQQQQQQPQD--------------
A. aegyp       ------------------------------------------------------------
D. magna       LPMAN--QQLQTNLTMPGARKQKLPVQQRRLANRQQQQQQQQQQQQQQQQQQQQQQQQQQ

D. pulex       ------QQQQQQHPPQQQLLGVLLNESSVQHTQGPQLSPGALQIMDDLLNAIPPNMTISR
A. aegyp       ------------------------------------------------------------
D. magna       SSSQEQQQQQQQPAPQQQLLGVLLNESSVQHTQGAQLSPGALQIMDDLLNAIPPNMTISR

D. pulex       SDSQVSPNYGSVMLSSPLGSGGPLASQISPSQRPMQPPFSPHGPMNTANGGNYNQSPQAQ
A. aegyp       ------------------------------------------------------------
D. magna       SDSQVSPNYGSVMLSSPLGS-GPLSSQISPGQRPMQPPFSPHGPMSAANGGNYNQNPQAQ

D. pulex       IVNNNSGNNFAPGGPSSRLSPFNSAQVSPRIGPPPQNFQQQQQQQQQQQHVSQLSPQPG
A. aegyp       ------------------------------------------------------------
D. magna       MANNSSSNNYAPGGPSSRLSPFSSAQVSPRIGAPSQGFQQQQQQQQQQQHGAQLSPQPG

D. pulex       VGARSSPGISSMQGQPSPGVGVGSWNNNMPNRPNLQPQQQRQQQ----------------
A. aegyp       ------------------------------------------------------------
D. magna       VGARSSPGIS-LQGQPSPGVAVGPWNNNVPNRPNLQPQRQQQQQQQQQPQQQQKQQQQQQ

D. pulex       -QQQPQQQPQQQHLLGYGQGIRGGYAPRPAMRALPNSGPPNCVGGPAGPGNSSNNNNNIR
A. aegyp       ------------------------------------------------------------
D. magna       QQQPPQPQPQQQHLLGYGQGIRSGYAPRPAIRSLANSGPPNCVGPPGPSS-----GNSNR

D. pulex       QGYGNNGMTDNPTGPSSSNGMFARQQQQQQQQQ-QQQQQQQQLRLQRTVSAPSGVIPGSV
A. aegyp       ------------------------------------------------------------
D. magna       QSYNNNGISDNPAGPNPSNGIFVRQQQQQQQQQQQQQQQQQQLRLQRTVSAPSGVIPALS

D. pulex       ------------------------------------------------------------
A. aegyp       ------------------------------------------------------------
D. magna       IGGGLSHNASNCVTNASVGSFRQHCREDVSTTSLHPSAPSALIHHHPPQPNQPPPPPPPP

D. pulex       ------------------------------------------------------------
A. aegyp       ------------------------------------------------------------
D. magna       PPAPPAPPAPPAPPSNSSSSSPYPSSQQSPHPSSPGYHLGMGNAGQLYQHQGVGGGGNGG

D. pulex       ------------------------------------------------------------
A. aegyp       ------------------------------------------------------------
D. magna       SGGSGSRGSASSAAAASVYSAKVLAFQQGSNRLAPPTSLGHPSGSQSSRPSSADDAVESF

D. pulex       ------------------------------------GGPVQGG----GRNDGGPPGMAS
A. aegyp       ------------------------------------------------------------
D. magna       FHFDSISSHQTCQHSHHLPFPPQGPLHPSELQLAGAGGGPVQVVGRNDGGNSGGGPAMAS
```

```
D. pulex       EYVRKELKAVVGARTIQRGLSGPVVNVN--HASGQMNHSSPPMMVPGGKPLNQADLEALG
A. aegyp       ------------------------------------------------------------
D. magna       EYVRKELKAVVGARTIQRGLSGPIGNLNQNLGSGPMNHSSPPMMVPGGKPLNQADLEALG

D. pulex       LSYELPQGNDGLHGRVWDSPNMGESPSQTMPSTRNTMEEAPRPADPQMSLLKQLLSE-
A. aegyp       ---------------------------------------------------------
D. magna       LSYELPQGNDGLHGRIWDSPNMGESPSQTMPSVRNTMEEAPRPADPQMSLLKQLLSE-
```
